# Effects of Establishment-Water Supply Method and Phosphorus Fertilizer Management on Growth, Soil Water Dynamics, and Grain Yield of Drip-Irrigated Winter Wheat

**DOI:** 10.3390/plants15192904

**Published:** 2026-09-23

**Authors:** Jiantao Ma, Shihui Nie, Yanting Zhang, Chunwu Wang, Chuanxin Chen, Juan Li, Yuting Hou, Haiyan Bi, Yongqiang Zhang, Junjie Lei

**Affiliations:** 1 Institute of Crop Research, Xinjiang Academy of Agricultural Sciences/Key Laboratory of Desert-Oasis Crop Physiology, Ecology and Cultivation, Urumqi 830091, China; mjt0323@163.com (J.M.); nieshihui@hotmail.com (S.N.); lixp618@163.com (C.C.); l_juan@yeah.net (J.L.); yuthou@163.com (Y.H.); 2 College of Agronomy, Xinjiang Agricultural University, Urumqi 830052, China; 18999027051@163.com; 3 Xinjiang General Station for Agricultural Technology Extension, Urumqi 830001, China; wcw9b612@163.com (C.W.); bihaiyan1026@163.com (H.B.)

**Keywords:** dry sowing followed by drip irrigation for emergence, split phosphorus fertigation, soil water, water-use efficiency, grain yield

## Abstract

Phosphorus (P) management may interact with the method used to supply water for crop establishment in drip-irrigated winter wheat. A two-year field experiment (2023–2025) in northern Xinjiang compared pre-sowing irrigation (F) with dry sowing followed by drip irrigation for emergence (D) under three P regimes: 100% basal P (P1), 70% basal + 30% fertigated P (P2), and 50% basal + 50% fertigated P (P3). Across the years, D increased the mean 0–100 cm soil water content by 15.76% relative to F. Under D, P3 increased the soil water content by 12.53% and maturity dry matter per plant by 31.02% relative to P1. DP3 produced the highest two-year mean grain yield (8416.2 kg ha^−1^), which was 29.60% higher than DP1 and 33.12% higher than FP1, and also achieved the highest water-use efficiency (WUE). The significant emergence-water method × P-management interaction for yield and yield components indicated that the response to split P application depended on the establishment-water regime. Random-forest analysis identified kernels per spike, spike number, 1000-grain weight, and soil water content as the principal predictors of yield variation. Under the conditions of this two-year single-site study, dry sowing followed by drip irrigation for emergence combined with 50% basal P + 50% fertigated P improved soil water availability, late-season biomass accumulation, grain yield, and WUE; multi-site validation is required before broader recommendation.

## 1. Introduction

In 2023, wheat occupied 1.21 million ha in Xinjiang, produced 7.03 million t of grain, and averaged 5.81 Mg ha^−1^ [1]. Winter wheat is a major cereal in northern Xinjiang and is grown within irrigated cereal rotations. Drip irrigation and fertigation have expanded as water-saving production practices, but their performance depends on synchronizing seedbed water supply with nutrient timing.

Water scarcity remains a major constraint on stable wheat production in arid and semiarid regions. Productive use of limited irrigation water depends on the amount and timing of water input, its distribution within the root zone, and its synchronization with crop development [2,3,4]. Appropriate irrigation can increase grain yield and WUE by limiting nonproductive water loss and maintaining water supply during critical growth stages [5,6,7]. However, high WUE is agronomically useful only when it is accompanied by adequate grain yield.

Phosphorus is essential for energy metabolism, membrane formation, carbohydrate transport, and crop establishment [8,9,10]. Because inorganic P is poorly mobile and readily adsorbed or precipitated in many soils, its availability depends strongly on fertilizer placement, timing, soil chemistry, root activity, and the distribution of water in the rhizosphere [11,12,13]. Improving P-use efficiency therefore requires better spatial and temporal matching between P supply and crop demand [14,15,16] while maintaining the long-term soil P pool [17,18]. General nutrient-efficiency frameworks [19,20,21] and drip irrigation research further emphasize management of the nutrient source, rate, timing, placement [22,23], and localized wetting. Drip fertigation can apply soluble nutrients within a localized wetted zone [24,25], but the effectiveness of split P supply may depend on how establishment water shapes that zone.

The wheat grain yield is determined by spike number, kernels per spike, and grain weight, and is supported by both remobilized pre-anthesis reserves and post-anthesis assimilation [26,27]. Accordingly, water–P management should be evaluated through linked responses in soil water, plant growth, dry-matter accumulation, yield components, grain yield, and water-use efficiency rather than through yield alone.

Field studies on winter wheat, including recent drip-fertigation experiments, show that irrigation amount, irrigation frequency, and fertilizer rate jointly regulate soil water extraction, post-anthesis dry-matter accumulation, yield components, grain yield, and resource-use efficiency [28,29]. Studies focused on P management further indicate that split or optimized P supply can improve P accumulation, crop growth, and yield formation, although responses depend on soil properties, temperature conditions, and the accompanying N and K regimes [30]. However, evidence remains limited on whether the method used to supply water for crop establishment modifies the response of winter wheat to split P fertigation under the arid conditions of northern Xinjiang.

This study therefore addressed three questions: (1) how pre-sowing irrigation and dry sowing followed by drip irrigation for emergence alter the temporal and vertical distribution of soil water; (2) whether the response to 30% or 50% split P fertigation depends on the establishment-water regime; and (3) how these treatments affect biomass accumulation, yield components, grain yield, WUE, and irrigation water-use efficiency (IWUE). The novelty lies in jointly evaluating establishment-water supply and P-application timing over two seasons in drip-irrigated winter wheat while linking soil water dynamics with crop growth and yield formation.

## 2. Results

### 2.1. Effects of Emergence-Water Supply Method and P-Application Strategy on Soil Water Content

#### 2.1.1. Mean Soil Water Content in the 0–100 cm Profile at Different Growth Stages

The mean soil water content in the 0–100 cm profile varied with growth stage and year (Figure 1). In 2023–2024, it peaked at booting, declined at anthesis, and increased again from grain filling to maturity; in 2024–2025, it declined continuously after jointing. Across the growing season, D exceeded F by 28.90% in 2023–2024 and by 2.86% in 2024–2025, giving a two-year mean increase of 15.76%.

Within both establishment-water regimes, the soil water content generally increased as P application shifted from a single basal application to split fertigation. Across years, P2 and P3 exceeded P1 by 7.54% and 12.53%, respectively, under D and by 15.21% and 25.10% under F. DP3 maintained the highest or near-highest values at most middle and late growth stages.

#### 2.1.2. Mean Soil Water Content at Different Soil Depths

Across treatments, the soil water content increased to a relative maximum near 60 cm and then declined toward 100 cm (Figure 2). The two-year mean at 60 cm was 19.13%, exceeding the corresponding values at 20, 40, 80, and 100 cm by 10.79%, 7.89%, 8.24%, and 13.98%, respectively, which identifies 40–80 cm as the principal wetted zone under the experimental drip system.

The profile response varied between years. D generally maintained more water than F across depths in 2023–2024, whereas shallow-layer differences narrowed in 2024–2025. DP3 nevertheless retained a relatively high water content in the 40–100 cm layers. Across the profile and both years, the mean soil water content was 20.05% under DP3, which was 12.53% higher than under DP1 and 38.51% higher than under FP1.

#### 2.1.3. Spatiotemporal Distribution of Soil Water Content Across Growth Stages and Soil Layers

The heatmaps show distinct interannual patterns in the 0–100 cm profile (Figure 3). Soil water changed little before jointing in both seasons. Thereafter, it recovered at grain filling in 2023–2024 but declined continuously in 2024–2025. Across treatments, the main wetted zone was concentrated at 40–80 cm.

The advantage of D over F was widespread across stages and layers in 2023–2024 but emerged mainly in the middle and deeper layers after jointing in 2024–2025. Within each establishment-water regime, P2 and P3 generally maintained more soil water than P1, and the DP3–DP1 difference at anthesis was 17.89% in 2023–2024 and 19.73% in 2024–2025.

Overall, DP3 showed the most persistent zone of relatively high water content in the middle soil profile after anthesis. This pattern was associated with, but does not by itself establish a causal mechanism for, the higher late-season biomass and yield observed under DP3.

### 2.2. Effects of Emergence-Water Supply Method and P-Application Strategy on Water Consumption and Water-Use Efficiency

Across all treatments, the mean grain yield increased from 7054.3 kg ha^−1^ in 2023–2024 to 7538.9 kg ha^−1^ in 2024–2025 (+6.9%). However, WUE and IWUE responses varied among treatments between years because these indices integrate grain yield and water consumption. The water-consumption components, WUE, and IWUE differed among treatments and years (Figure 4). Because WUE and IWUE incorporate grain yield, the corresponding yield data for each treatment and year are reported in Table 1. Averaged across years, IWUE and WUE under F were 7.51% and 8.08% lower, respectively, than under D; however, year-to-year changes were not uniform among individual treatments.

Within each establishment-water regime, soil water consumption generally followed P1 > P2 > P3, whereas IWUE and WUE followed P3 > P2 > P1. DP3 had the highest two-year mean WUE and IWUE. These values reflect the combined treatment effects on grain yield and the water-balance denominator.

### 2.3. Effects of Emergence-Water Supply Method and P-Application Strategy on Plant Growth and Dry-Matter Accumulation

#### 2.3.1. Plant Height

Plant height increased with crop development, and treatment differences became clearer after jointing (Figure 5). Across the years, the mean height at grain filling was highest under DP3 (78.18 cm) and lowest under FP1 (69.50 cm). DP3 exceeded DP1 and FP1 by 7.15% and 12.49%, respectively, whereas DP2 and FP3 showed intermediate responses.

#### 2.3.2. Dry-Matter Accumulation per Plant

Dry matter per plant increased throughout crop development, and treatment differences widened after anthesis (Figure 6). In both years, DP3 generally maintained more dry matter than DP1 and FP1 across the measured stages.

At maturity, the two-year mean dry matter per plant was highest under DP3 (6.33 g), 31.02% and 35.17% higher than under DP1 and FP1, respectively. The larger treatment differences late in the season indicate that the biomass response was expressed mainly after anthesis; physiological mechanisms were not measured directly.

### 2.4. Effects of Emergence-Water Supply Method and P-Application Strategy on Grain Yield and Yield Components

Grain-yield ranking was consistent in both years: DP3 > DP2 > FP3 > FP2 > DP1 > FP1 (Table 1). DP3 produced a two-year mean of 8416.2 kg ha^−1^, 29.60% higher than DP1 and 33.12% higher than FP1. The mean yield across all treatments was 6.9% higher in 2024–2025 than in 2023–2024. Grain-yield ranking was consistent in both years: DP3 remained the highest-yielding treatment and FP1 the lowest. The mean yield increased from 7054.3 kg ha^−1^ in 2023–2024 to 7538.9 kg ha^−1^ in 2024–2025 (+6.9%).

The emergence-water supply method and P-application strategy had highly significant main effects on spike number, kernels per spike, 1000-grain weight, and grain yield. Relative to DP1, DP3 increased the two-year means of these three yield components by 20.85%, 11.72%, and 5.75%, respectively. The significant emergence-water method × P-management interaction for all four variables indicates that the response to split P application depended on the establishment-water regime.

### 2.5. Key Drivers of Grain Yield and Water-Use Efficiency

The random-forest analysis identified kernels per spike as the most important predictor of grain-yield variation, followed by spike number, 1000-grain weight, and soil water content; the model had R^2^ = 0.69 (Figure 7). Plant height and dry matter per plant were not significant predictors in this model. Variable importance represents statistical predictive contribution and should not be interpreted as proof of causation.

In the WUE model, grain yield had the highest importance, while soil water content and 1000-grain weight were also significant; the model had R^2^ = 0.75. Correlation and Mantel analyses described associations among soil water, crop traits, grain yield, and WUE. Together, these analyses indicate that improvements in WUE occurred when the proportional gain in yield exceeded the corresponding change in water consumption.

## 3. Discussion

### 3.1. Soil Water and Water-Use Efficiency Responses to Establishment-Water Supply and Split P Application

Crop WUE is jointly determined by grain yield and water consumption; consequently, a treatment can improve the WUE by increasing yield, reducing water use, or both [2,3,4]. In this study, D was associated with a higher mean 0–100 cm soil water content, WUE, and IWUE than F, but the size of the difference varied markedly between seasons. This pattern is consistent with reports that irrigation timing and frequency alter soil water extraction and yield formation in drip-fertigated winter wheat [31,32,33]. Because the total crop-establishment irrigation differed between F and D, the observed response should be interpreted as the combined effect of water supply timing and amount rather than timing alone [34,35].

P3 was associated with a lower calculated soil water consumption and higher WUE and IWUE than P1. One plausible explanation for this is that split P supply altered the temporal coincidence of nutrient availability and crop water demand within the wetted soil volume [36,37]. However, P movement, root distribution, plant P uptake, evaporation, and deep percolation were not measured; the proposed water–P coordination therefore remains a hypothesis based on soil water patterns and prior literature [38,39]. The much larger D–F difference in 2023–2024 than in 2024–2025 further shows that treatment performance was sensitive to annual weather and initial soil water conditions. Because WUE is the ratio of yield to evapotranspiration, differing year-to-year changes in its numerator and denominator also explain why WUE increased for some treatments but decreased for others.

### 3.2. Plant Growth and Dry-Matter Responses to Establishment-Water Supply and Split P Application

Treatment differences in plant height and dry matter per plant became clearer after jointing, and DP3 maintained the highest late-season values. This association is compatible with improved synchronization of soil water availability and nutrient supply, but the present measurements do not identify the physiological pathway. In particular, crop emergence, root architecture, leaf area, photosynthetic rate, and plant P uptake were not measured.

In calcareous soils, P diffusion is limited and applied P can be adsorbed or precipitated by Ca-containing components [9,12]. Applying part of the P fertilizer with winter irrigation could therefore have changed the location and timing of P availability within the wetted zone [30,36,40]. Nevertheless, the present study did not measure soil P distribution or root zone P availability, so statements about P movement toward active roots are presented as plausible explanations rather than demonstrated mechanisms.

The year-specific timing of treatment differences indicates that plant height and biomass responses were also influenced by meteorological conditions and initial soil moisture [41]. The late-season biomass advantage under DP3 may have supported grain filling, but confirmation will require direct measurements of canopy development, gas exchange, root traits, and P accumulation. The current results therefore demonstrate treatment associations with crop growth rather than a specific physiological mechanism.

### 3.3. Grain-Yield Formation Under Establishment-Water Supply and Split P Application

The wheat grain yield is jointly determined by spike number, kernels per spike, and 1000-grain weight [42,43,44]. DP3 produced the highest yield in both seasons, and its advantage over DP1 was accompanied mainly by more spikes and kernels per spike, with a smaller increase in 1000-grain weight. The significant emergence-water method × P-management interaction indicates statistical synergy in the sense that the response to split P application depended on the establishment-water regime; it does not identify the developmental processes responsible.

Improved establishment moisture, tiller survival, floret development, and post-anthesis assimilation are biologically plausible explanations for the yield-component responses [45], but none were measured directly. Accordingly, the results should not be interpreted as evidence that D improved emergence uniformity or that split P reduced floret abortion. These mechanisms require targeted measurements of stand establishment, tiller dynamics, spike development, root growth, P uptake, and photosynthetic traits.

The random-forest analysis identified kernels per spike, spike number, and 1000-grain weight as important predictors of yield variation. This predictive ranking complements the treatment comparisons but does not establish causal order. Taken together, the ANOVA and random-forest results support an association among establishment-water supply, split P management, soil-water dynamics, yield components, and final yield, while leaving the underlying mechanisms to be tested in future experiments.

The profitability of DP3 will depend on irrigation energy, drip-system operation, fer-tilizer price, labor, and grain value. In addition, this experiment was conducted for two seasons at one sandy-loam site; validation across sites, soil textures, irrigation schedules, and climatic conditions is required before regional recommendation.

## 4. Materials and Methods

### 4.1. Experimental Site

The experiment was conducted from September 2023 to July 2025 at the Qitai Wheat Experimental Station of the Xinjiang Academy of Agricultural Sciences (89°13′–91°22′ E, 42°25′–45°29′ N; 895 m above sea level). The region has a temperate continental arid-to-semiarid climate, with a mean annual temperature of 7.1 °C, a mean July temperature of 22.6 °C, an extreme maximum temperature of 38.9 °C, a mean annual relative humidity of 55%, a frost-free period of approximately 153 d, and mean annual precipitation of 191.10 mm. The experimental soil was sandy loam. In the 0–20 cm layer, soil pH was 8.72, organic matter was 13.90 g kg^−1^, total salts were 0.58 g kg^−1^, total N was 1.18 g kg^−1^, total P was 1.30 g kg^−1^, total K was 23.43 g kg^−1^, alkali-hydrolyzable N was 40.56 mg kg^−1^, available P was 38.65 mg kg^−1^, and available K was 193.58 mg kg^−1^. Air temperature and precipitation during the two wheat-growing seasons are shown in Figure 8.

### 4.2. Experimental Design

A two-factor split-plot design was used. The main-plot factor was establishment-water supply method, including pre-sowing irrigation (F) and dry sowing followed by drip irrigation for emergence (D). For the F treatment, pre-sowing irrigation was applied 10–15 days before land preparation at 1200 m^3^ ha^−1^. For the D treatment, seeds were sown under dry conditions and drip irrigation was immediately applied after sowing; the first irrigation supplied 600 m^3^ ha^−1^, followed by a second irrigation of 300 m^3^ ha^−1^ 3–5 days later. The subplot factor was the phosphorus (P) application strategy. Under the same total P input, three P management treatments were established: 100% basal application (P1), 70% basal application + 30% drip fertigation (P2), and 50% basal application + 50% drip fertigation (P3). The total diammonium phosphate application rate was 172.5 kg ha^−1^. The basal P fertilizer was incorporated into the soil before sowing, and the fertigated P fraction was supplied with irrigation water. The winter wheat cultivar ‘Xindong 22’ was manually sown on 20 September 2023 at a seeding rate of 6.0 × 10^6^ seeds ha^−1^ with 20 cm row spacing. Each plot covered 12 m^2^ (2 m × 6 m), and each treatment was replicated three times.

The preceding crop was winter wheat for both experimental seasons (2023–2024 and 2024–2025).

During the winter wheat growing seasons, winter irrigation was applied on 15 October 2023 and 28 October 2024, with an irrigation amount of 900 m^3^ ha^−1^ in each year. After spring regrowth, water and nutrient management followed the local high-standard field management practice. Therefore, the irrigation events described above were consistent with the original field records.

### 4.3. Measurements

#### 4.3.1. Soil Water Content, Water Consumption, and Water-Use Efficiency

Volumetric soil water content was measured at spring regrowth, jointing, booting, anthesis, grain filling, and maturity using a TRIME-PICO soil-moisture meter (IMKO Micromodultechnik GmbH, Ettlingen, Germany) in the 0–20, 20–40, 40–60, 60–80, and 80–100 cm layers, with three measurement points per plot. Soil water storage in each layer was calculated from volumetric water content and layer thickness, and water storage in the 0–100 cm profile was calculated as the sum of all layers. Assuming negligible surface runoff and deep percolation, seasonal evapotranspiration was calculated using the water-balance equation:

ET = I + P + (SWSpre-sowing − SWSmaturity)

where ET is total water consumption during the growing season (mm), I is irrigation (mm), P is effective precipitation during the growing season (mm), and SWS is soil water storage in the 0–100 cm profile (mm). Water-use efficiency was calculated as WUE = grain yield/ET, and irrigation water-use efficiency as IWUE = grain yield/I.

#### 4.3.2. Plant Height and Dry Matter per Plant

At jointing, booting, heading, anthesis, and grain filling, 10 uniformly growing plants were randomly selected from each plot, and height from the soil surface to the top of the spike (excluding awns) was measured. Representative plants were sampled at jointing, booting, anthesis, grain filling, and maturity and separated into leaves, stems plus sheaths, and spikes; at maturity, samples were separated into grain and non-grain organs. Samples were heated at 105 °C for 30 min to halt metabolic activity and then oven-dried at 80 °C to constant mass to calculate dry matter per plant.

#### 4.3.3. Grain Yield and Yield Components

At maturity, spike number was counted in two adjacent 1 m rows in each plot, and kernels per spike were determined from 10 representative plants. Grain was harvested from a 1 m^2^ area in each plot. Grain moisture was measured, and yield and 1000-grain weight were standardized to the reference moisture content.

### 4.4. Statistical Analysis

Data were organized in Microsoft Excel 2016 and analyzed using analysis of variance in SPSS 26.0. Year (Y), emergence-water method (DF), and P-application strategy (P) were treated as fixed factors in a three-way ANOVA. When an interaction was significant, simple effects were analyzed separately by year. Treatment means were compared using Duncan’s multiple-range test at *p* < 0.05. Figures were prepared in Origin 2024. The random-forest variable-importance analysis, Pearson correlation analysis, and Mantel tests were performed in R 4.5.3 (R Foundation for Statistical Computing, Vienna, Austria; https://www.r-project.org/, accessed on 12 September 2026). Variable importance for grain yield and WUE was quantified using the percentage increase in mean squared error (%IncMSE). Data in figures and tables are presented as means ± standard errors.

## 5. Conclusions

Under the conditions of this two-year single-site experiment, dry sowing followed by drip irrigation for emergence combined with 50% basal P + 50% fertigated P (DP3) produced the highest grain yield and WUE. Its performance was associated with higher soil water content in the 0–100 cm profile, greater late-season dry matter per plant, more spikes per unit area, and more kernels per spike. The significant emergence-water method × P-management interaction indicates that the response to split P fertigation depended on the establishment-water regime. DP3 may therefore be considered a promising option for drip-irrigated winter wheat on comparable sandy-loam soils in northern Xinjiang, but confirmation across additional sites and years, together with full-season irrigation, nutrient-balance, and economic analyses, is required before regional recommendations.

## Figures and Tables

**Figure 1:**
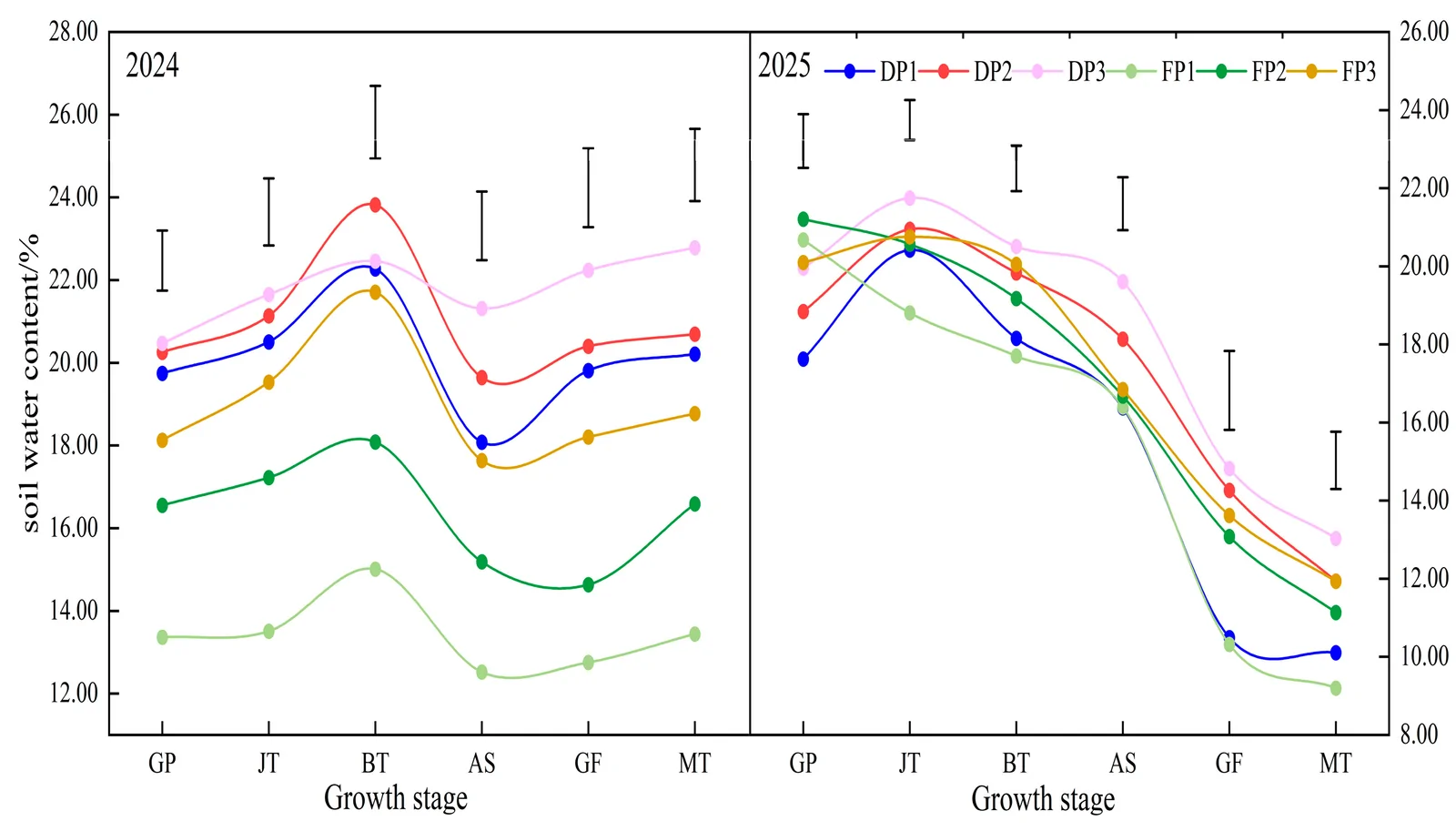
Mean soil water content in the 0–100 cm profile at different growth stages under different treatments. Note: GP, getting up; JT, jointing; BT, booting; AS, anthesis; GF, grain filling; MT, maturity.

**Figure 2:**
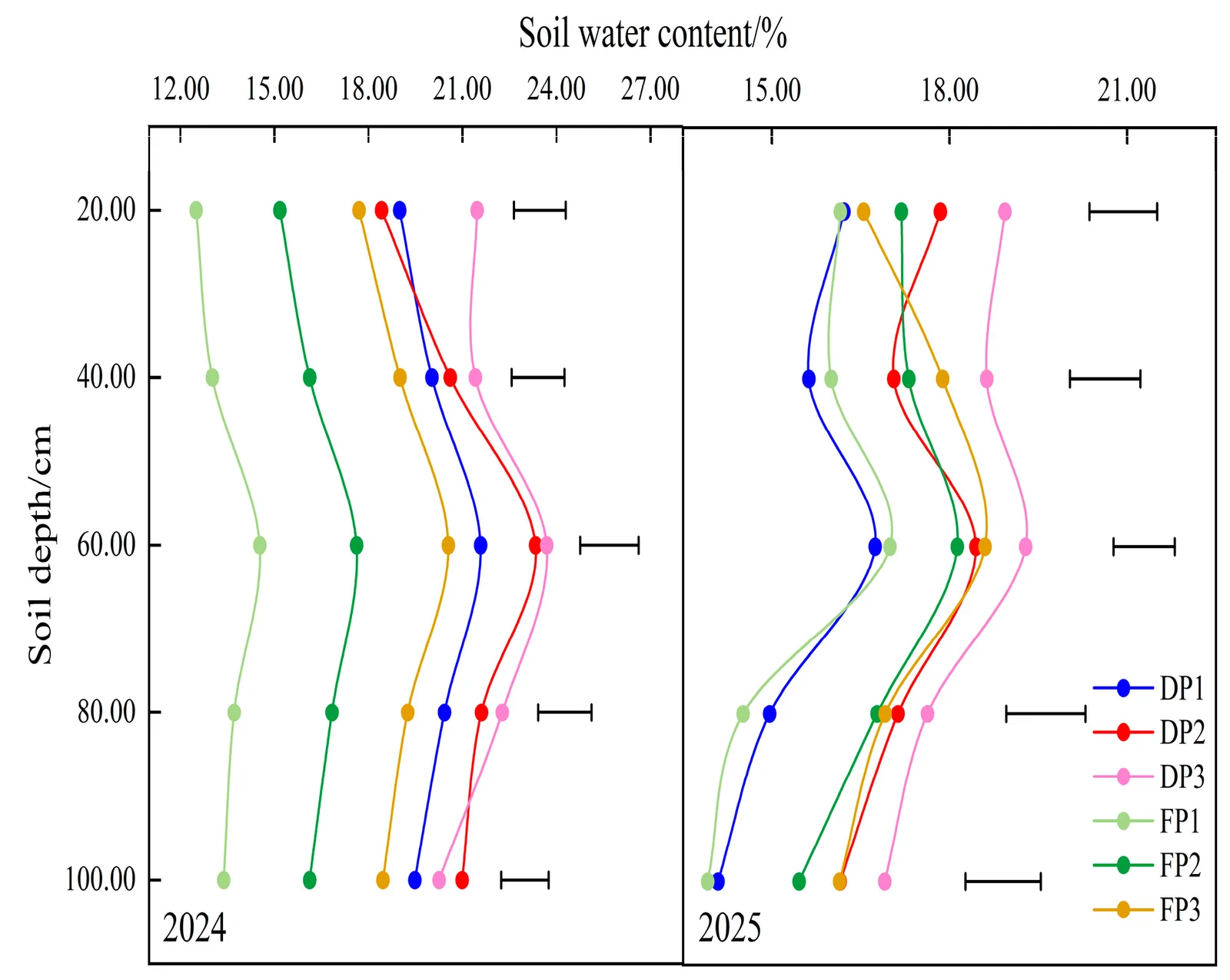
Mean soil water content at different soil depths over the entire growing season under different treatments.

**Figure 3:**
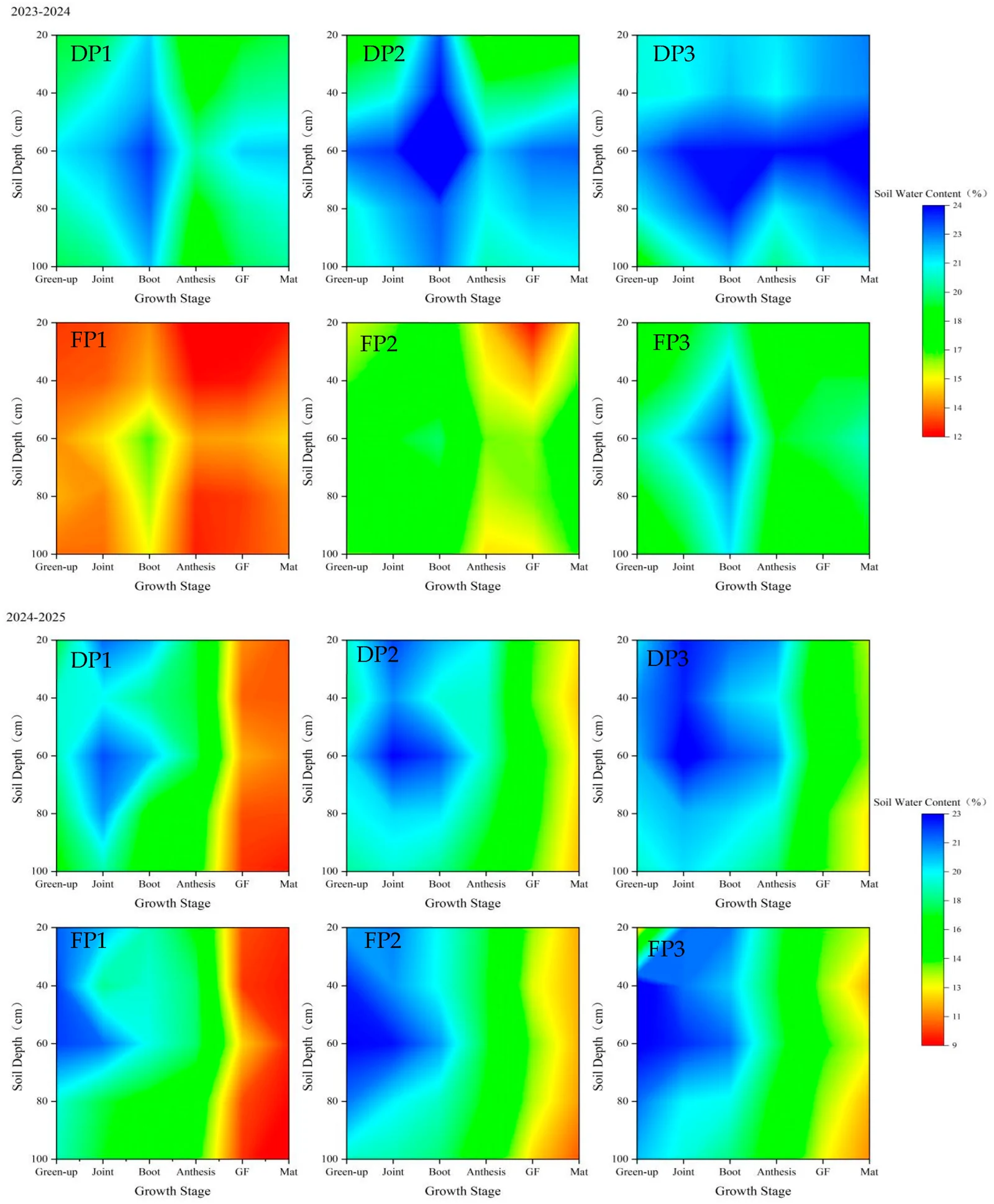
Spatiotemporal distribution of soil water content in the 0–100 cm profile under different treatments. Separate year-specific color scales were used to preserve within-year contrast; colors should therefore not be compared directly between years.

**Figure 4:**
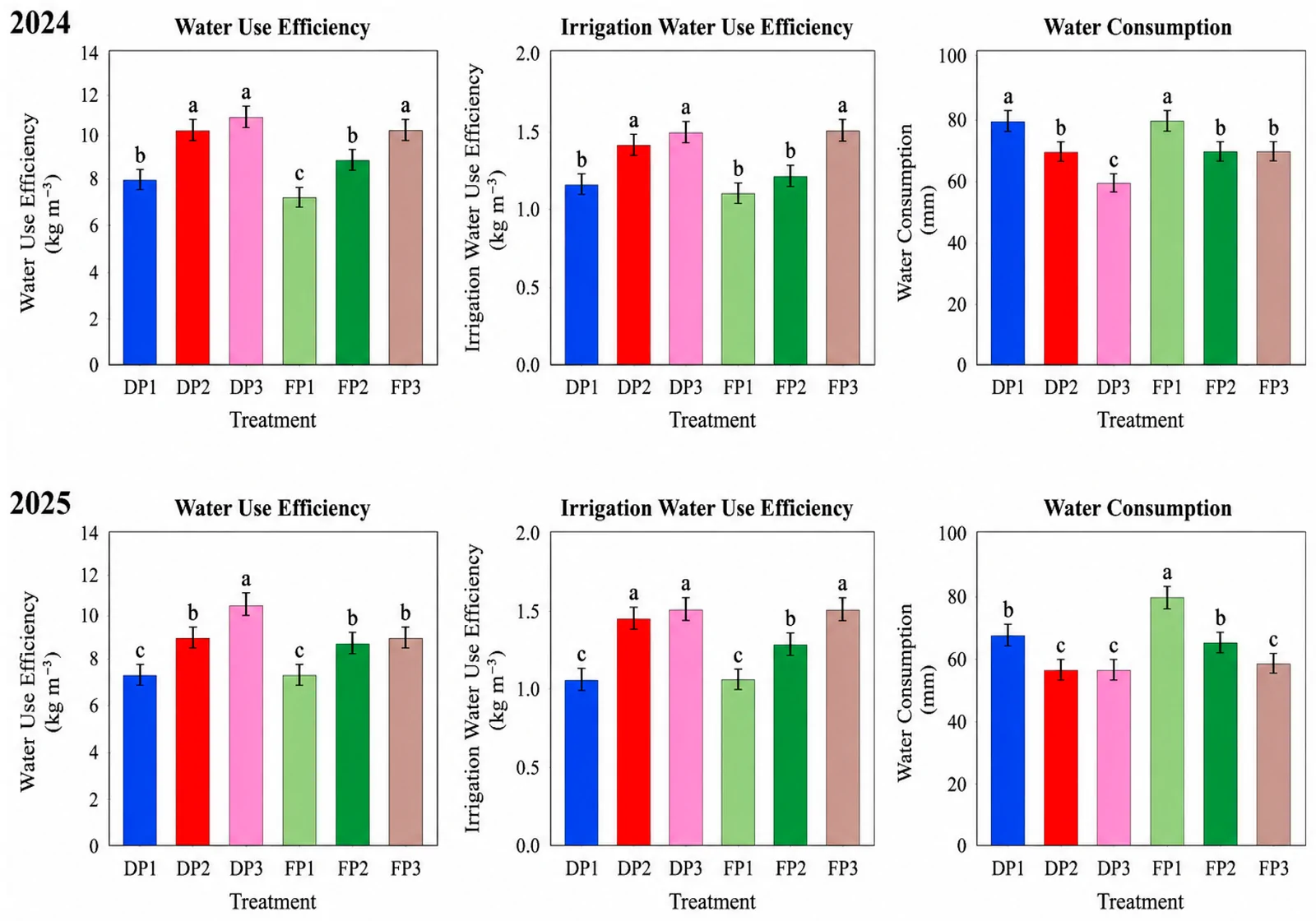
Water consumption, water-use efficiency (WUE), and irrigation water-use efficiency (IWUE) of winter wheat under different treatments. Different lowercase letters indicate significance within each year (Duncan’s test, *p* < 0.05).

**Figure 5:**
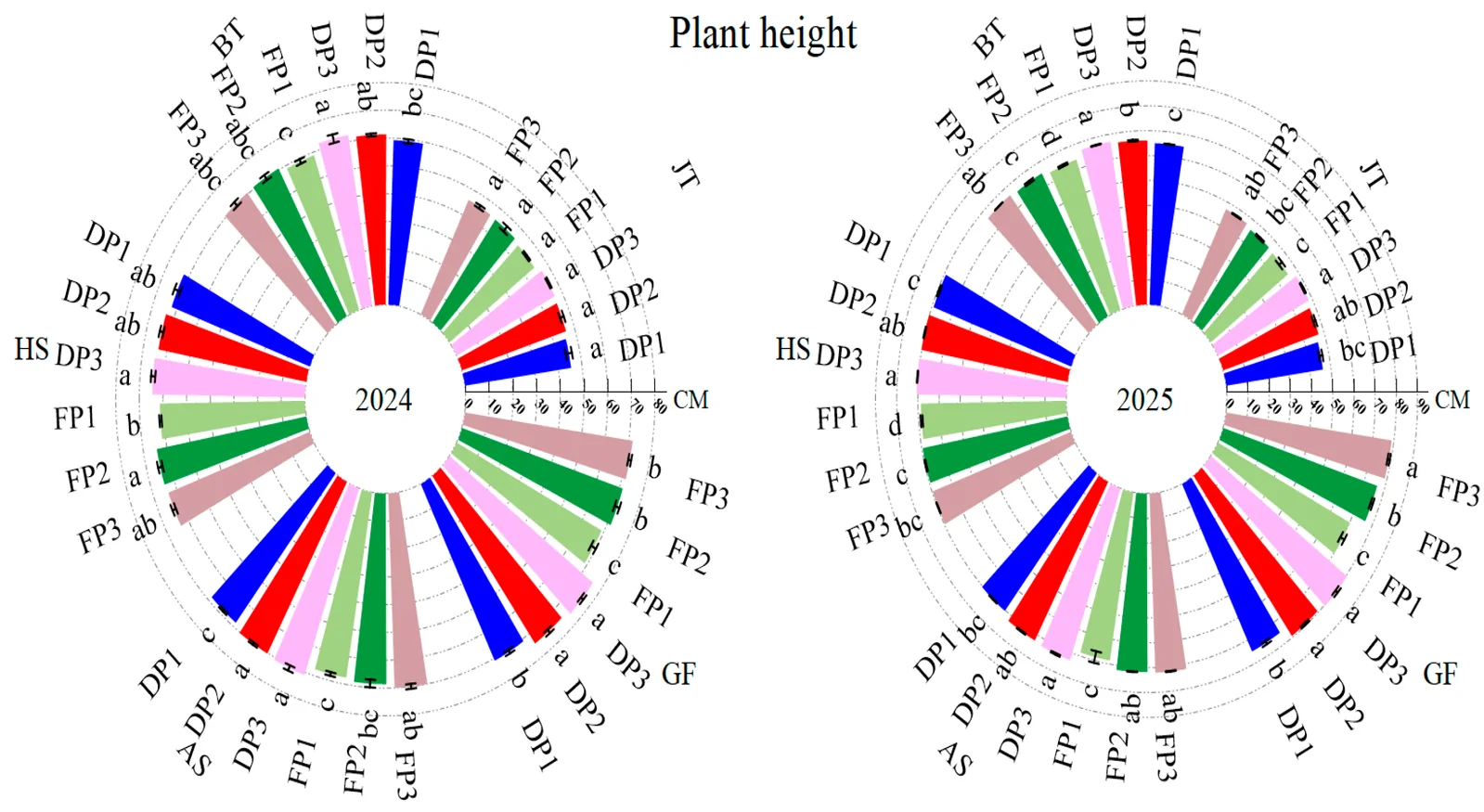
Plant height of winter wheat under different treatments. Note: JT, jointing; BT, booting; HS, heading stage; AS, anthesis; GF, grain filling. Different lowercase letters indicate significance within each growth stage and year (Duncan’s test, *p* < 0.05).

**Figure 6:**
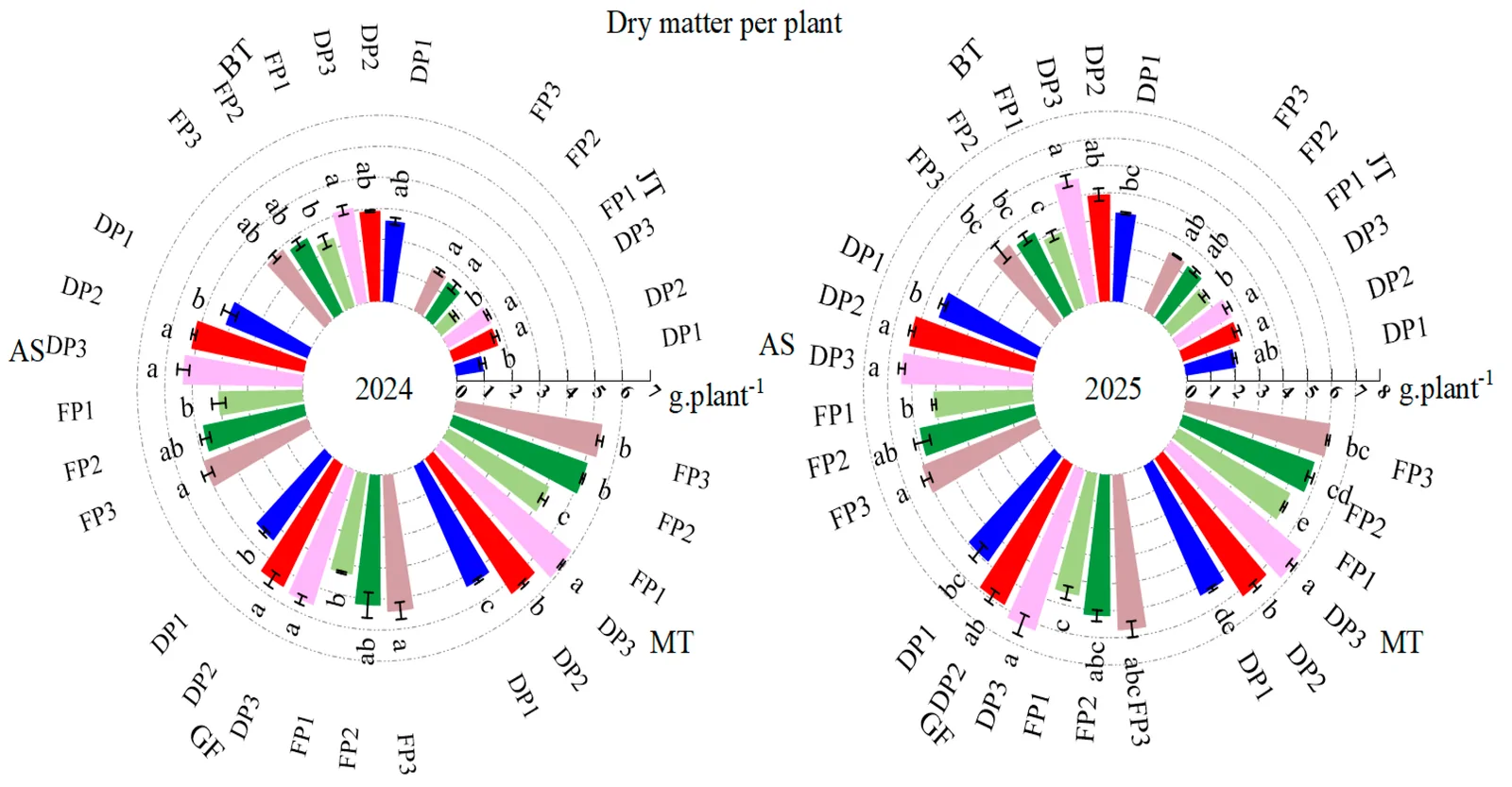
Dry matter per plant of winter wheat under different treatments. Note: JT, jointing; BT, booting; AS, anthesis; GF, grain filling; MT, maturity. Different lowercase letters indicate significance within each growth stage and year (Duncan’s test, *p* < 0.05).

**Figure 7:**
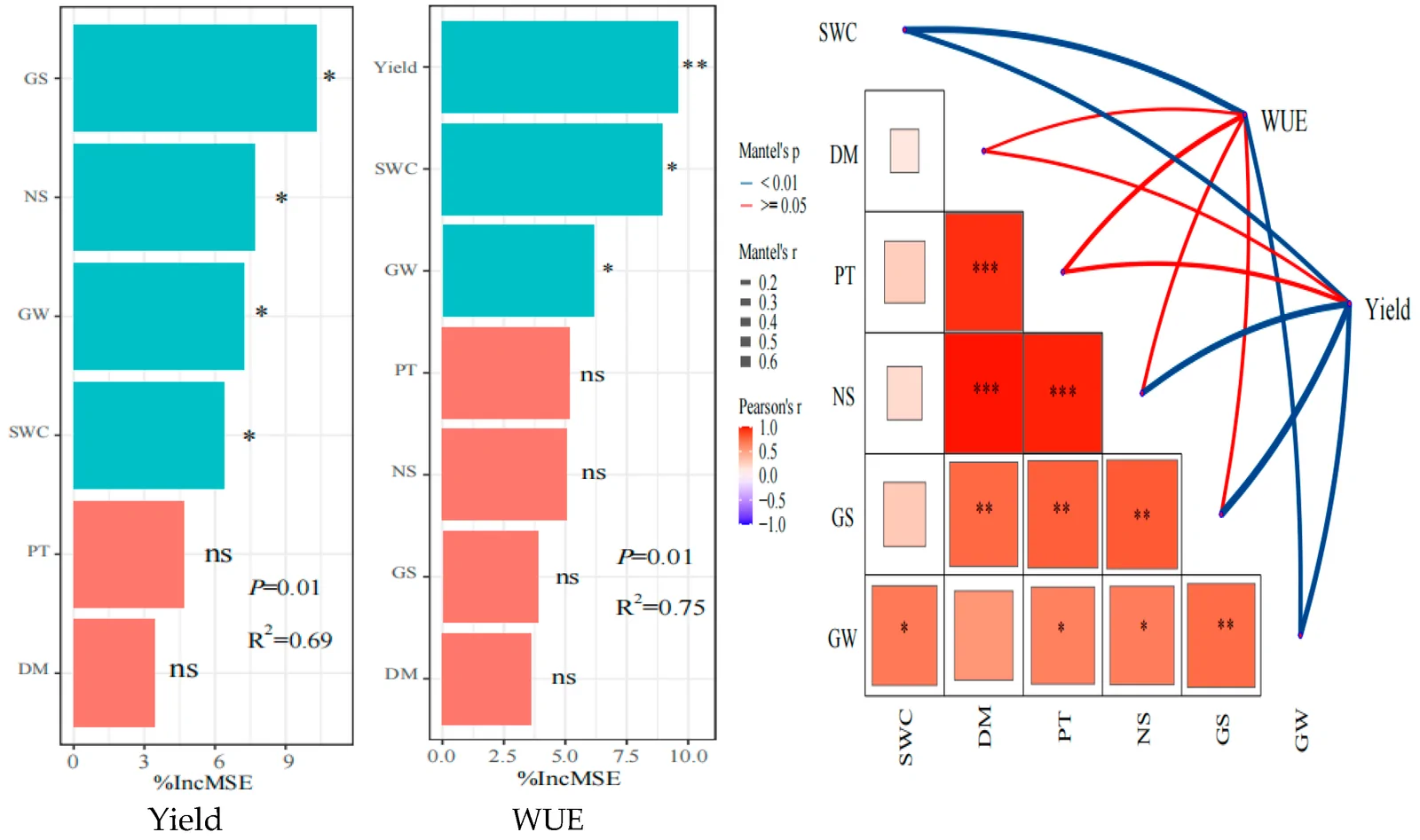
Random-forest variable importance and correlation network for grain yield and water-use efficiency. Note: SWC, soil water content; DM, dry matter per plant; PT, plant height; NS, spike number; GS, kernels per spike; GW, 1000-grain weight. * *p* < 0.05; ** *p* < 0.01; *** *p* < 0.001; ns, not significant.

**Figure 8:**
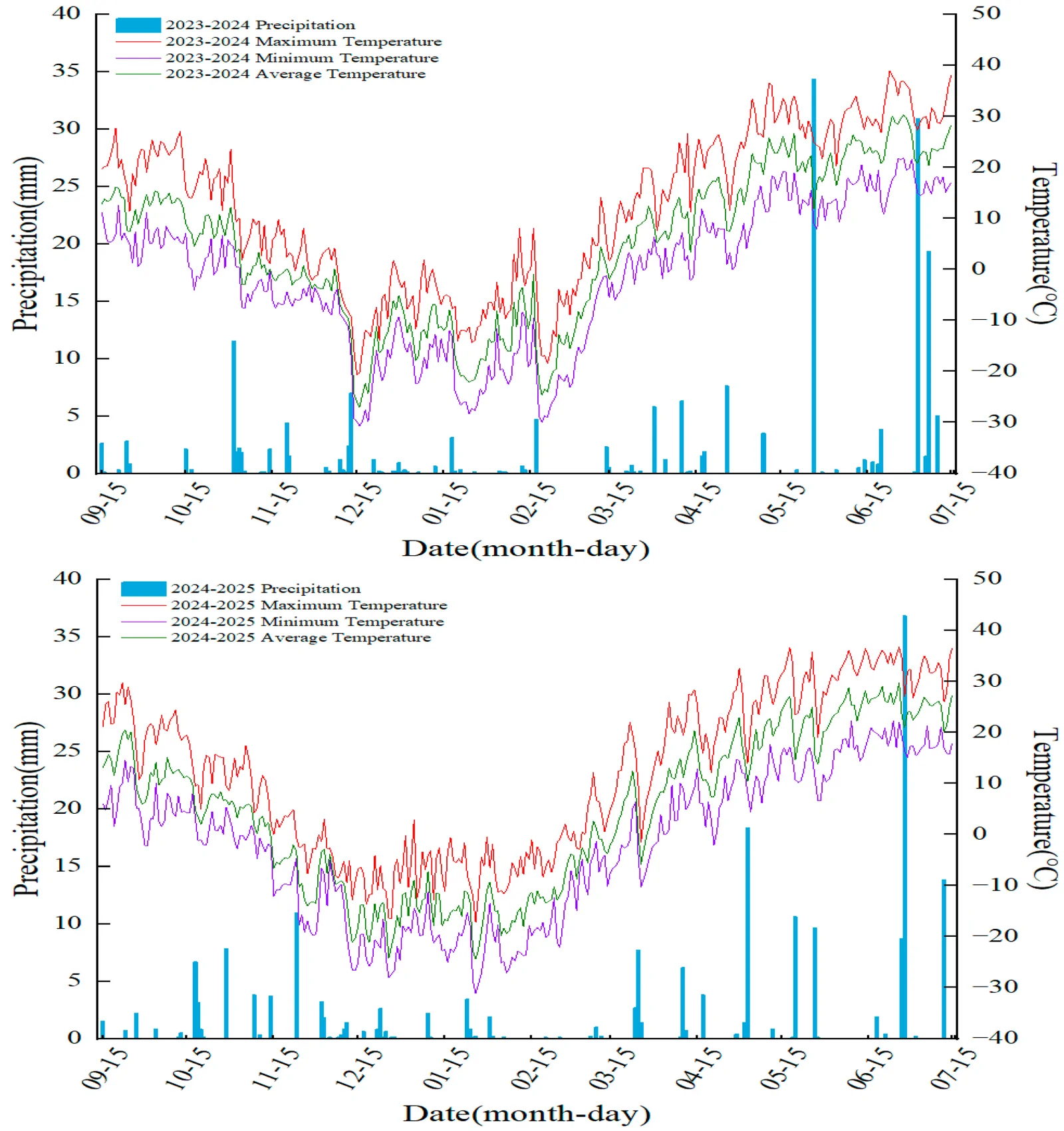
Meteorological conditions during the 2023–2024 and 2024–2025 winter wheat growing seasons.

**Table 1:** Grain yield and yield components of winter wheat under different treatments.

Year	Treatment	Spike Number (×10^4^ ha^−1^)	Kernels per Spike	1000-Grain Weight (g)	Grain Yield (kg ha^−1^)
2023–2024	DP1	572.0 ± 18.5 d	32.8 ± 0.5 b	39.2 ± 0.6 bc	6355.0 ± 170.5 c
	DP2	647.7 ± 12.3 b	34.9 ± 0.6 a	40.3 ± 0.3 b	7671.9 ± 120.1 b
	DP3	668.3 ± 21.7 a	35.1 ± 0.1 a	41.7 ± 0.2 a	8032.4 ± 132.8 a
	FP1	564.3 ± 13.4 d	33.7 ± 1.2 ab	38.9 ± 0.8 c	6277.8 ± 189.6 c
	FP2	611.7 ± 12.3 c	33.9 ± 0.3 ab	39.5 ± 0.3 bc	6514.4 ± 94.7 c
	FP3	602.3 ± 7.4 c	34.3 ± 0.4 ab	39.3 ± 0.3 bc	7474.4 ± 125.1 b
2024–2025	DP1	631.7 ± 6.2 e	32.7 ± 0.4 d	39.0 ± 0.3 b	6633.3 ± 262.5 c
	DP2	728.3 ± 10.3 b	37.3 ± 1.1 ab	40.2 ± 0.3 ab	8066.7 ± 235.7 ab
	DP3	786.3 ± 7.4 a	38.1 ± 1.5 a	41.0 ± 1.2 a	8800.0 ± 294.4 a
	FP1	593.3 ± 15.5 f	32.6 ± 0.4 d	39.0 ± 0.4 b	6366.7 ± 205.5 c
	FP2	656.7 ± 6.2 d	34.0 ± 0.5 cd	39.4 ± 0.8 b	7466.7 ± 124.7 b
	FP3	688.3 ± 10.3 c	35.1 ± 1.1 bc	39.8 ± 0.5 ab	7900.0 ± 253.2 b
Emergence-water supply method (DF)		252.14 **	12.48 **	16.85 **	27.69 **
P-application strategy (P)		297.02 **	22.33 **	13.99 **	72.24 **
Y × DF		24.16 **	7.29 *	0.82	0.001
Y × P		25.99 **	4.72 *	0.02	1.82
DF × P		26.11 **	5.61 *	4.24 *	3.64 *
Y × DF × P		0.05	0.37	0.58	1.52

Note: Values are means ± SE (*n* = 3). Different lowercase letters within a year indicate significant differences among treatments (Duncan’s test, *p* < 0.05). * *p* < 0.05; ** *p* < 0.01. Y, year; DF, emergence-water supply method; P, P-application strategy. The lower block reports F-values from the three-way ANOVA.

## Data Availability

The original contributions presented in this study are included in the article. Further inquiries can be directed to the corresponding author.
